# Characterization of the *Sesbania rostrata* Phytochelatin Synthase Gene: Alternative Splicing and Function of Four Isoforms

**DOI:** 10.3390/ijms10083269

**Published:** 2009-07-24

**Authors:** An-Ming Li, Bing-Yun Yu, Fu-Hua Chen, Hui-Yan Gan, Jian-Gang Yuan, Rongliang Qiu, Jun-Chao Huang, Zhong-Yi Yang, Zeng-Fu Xu

**Affiliations:** 1 State Key Laboratory of Biocontrol and Key Laboratory of Gene Engineering of the Ministry of Education, School of Life Sciences, Sun Yat-sen University, Guangzhou 510275, China; E-Mails: lsahmp68@yahoo.com.cn (A.M.L.); ybyun96@yahoo.com.cn (B.Y.Y.); cyy-gz@163.com (F.H.C.); incredibly861@163.com (H.Y.G.); yjgstorm@163.com (J.G.Y.); 2 Laboratory of Molecular Breeding of Energy Plants, Xishuangbanna Tropical Botanical Garden, Chinese Academy of Sciences, Kunming 650223, Yunnan, China; 3 School of Environmental Science and Engineering, Sun Yat-sen University, Guangzhou 510275, China; E-Mail: eesqrl@mail.sysu.edu.cn (R.L.Q.); 4 School of Biological Sciences, The University of Hong Kong, Pokfulam Road, Hong Kong, China; E-Mail: drjunchao@yahoo.com (J.C.H.)

**Keywords:** alternative splicing, heavy metal tolerance, phytochelatin, phytochelatin synthase, phytoremediation, Sesbania rostrata

## Abstract

Phytochelatins (PCs) play an important role in detoxification of heavy metals in plants. PCs are synthesized from glutathione by phytochelatin synthase (PCS), a dipeptidyltransferase. *Sesbania rostrata* is a tropical legume plant that can tolerate high concentrations of Cd and Zn. In this study, the *S. rostrata* PCS gene (*SrPCS*) and cDNAs were isolated and characterized. Southern blot and sequence analysis revealed that a single copy of the *SrPCS* gene occurs in the *S. rostrata* genome, and produces four different *SrPCS* mRNAs and proteins, SrPCS1–SrPCS4, by alternative splicing of the *SrPCS* pre-mRNA. The SrPCS1 and SrPCS3 proteins conferred Cd tolerance when expressed in yeast cells, whereas the SrPCS2 and SrPCS4 proteins, which lack the catalytic triad and the N-terminal domains, did not. These results suggested that SrPCS1 and SrPCS3 have potential applications in genetic engineering of plants for enhancing heavy metal tolerance and phytoremediation of contaminated soils.

## 1. Introduction

Phytochelatins (PCs) have been found in all plant species investigated, a few fungal species and some marine diatoms [1]. PCs can act as high affinity metal chelators and facilitate the vacuolar sequestration of heavy metals, especially Cd^2+^ ions, and hence play in the plant kingdom an important role in detoxification of some heavy metals [2,3]. PCs have the general structure (γ-Glu-Cys)_n_-Gly, where n has been reported to be as high as 11, but generally ranges from 2 to 5 [4]; they are synthesized posttranslationally in the presence of heavy metal ions from glutathione (GSH) by PC synthase (PCS), a γ-glutamyl-cysteine transpeptidase [5]. So far, four different models for the catalytic mechanism of PCS have been proposed [5–10], although a consistent model that clearly demonstrates how PCS is activated and how PCs are synthesized has not been established. Alignment of PCS protein sequences reveals a high degree of similarity in the N-terminal domain, whereas the C-terminal domain which turns out to be extremely variable, is missing in the prokaryotic PCS proteins [11,12]. The N-terminal core domain of PCS, which can be referred to the catalytic domain sufficient to confer a PCS activity, has been confirmed by limited proteolysis and mutant analyses [6,8,11,13]. The variable C-terminal region was shown to ensure higher PCS activity, enhance protein stability and respond to a broader spectrum of heavy metals [11]. The stereostructure of the PCS from the cyanobacterium *Nostoc* sp. PCC7120 (NsPCS) in its native and γ-Glu-Cys-acylated condition was reported [14]. This study represented the first crystal structure of a PCS and established that prokaryotic NsPCS and the eukaryotic *Arabidopsis thaliana* PC synthase1 (AtPCS1) belong to the papain superfamily of cysteine proteases, with a conserved catalytic machinery that had been deduced from kinetic, protein chemical, and site mutagenic studies of the prototypical AtPCS1 [8,10,15].

PCS genes have been cloned and characterized in *Arabidopsis thaliana* (AtPCS1, [3,16]), *Schizosaccharomyces pompe* (SpPCS, [17]), *Triticum aestivum* (TaPCS1, [6]), *Lotus japonicus* (LjPCS, [18–20], the model nematode *Caenorhabditis elegans* (CePCS1, [21,22]) and the prokaryote *Nostoc* sp. PCC7120 (NsPCS, [8,12]). It has been proposed that PCS is a constitutive enzyme in general and its gene expression is not induced in response to heavy metal exposure [23]. This proposal was supported by the analysis of *AtPCS1* expression, which showed that the level of *AtPCS1* mRNA was unchanged on exposure to heavy metals such as Cd, Zn, or Cu, or to oxidative stress, salt stress, jasmonic acid or salicylic acid [6,10,24]. During the early stages of plant development, however, the steady-state level of *AtPCS1* mRNA increased by 2-fold in 5-day-old Cd-treated *Arabidopsis* compared to non-treated seedlings [25]. Clemens *et al*. [17] found that the level of *TaPCS1* in wheat roots treated with 100 μM Cd^2+^ was 5- to 10-fold higher than those of untreated roots. These studies indicated that in specific organs and developmental stages of plants, in which a strong defense against exposure to toxic heavy metals is required, the expression of the *PCS* genes may be regulated in response to heavy metals.

*Sesbania rostrata*, a legume native to tropical West Africa, is an annual species that bears stem nodules as well as root nodules associated with *Azorhizobium caulinodans*, a specialized fast-growing strain of *Rhizobia* that can fix nitrogen in its free-living form [26]. Yang *et al*. [27] found that *S. rostrata* can tolerate some heavy metals, especially Zn and Cd. To understand the mechanism of heavy metal tolerance in *S. rostrata*, in this study the corresponding PCS gene and its cDNAs were isolated and characterized.

## 2. Results and Discussion

### 2.1. Cloning and Sequence Analysis of PCS cDNAs and Gene from S. rostrata

**Figure 1.**
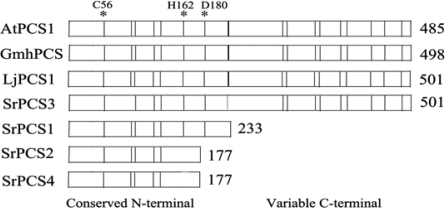
Schematic comparison of PCS polypeptides from different plants. The positions of the conserved Cys residues are indicated by vertical bars. They exhibit at least 60% identical amino acids in pair-wise comparison. The triad Cys-56, His-162 and Asp-180 are indicated as asterisks. AtPCS1, PCS from *A. thaliana*, (GenBank accession no. AAD41794); LjPCS1, PCS from *Lotus japonicus* (GenBank accession no. AY633847); SrPCS1-4, PCS1-4 from *S. rostrata* (GenBank accession nos. DQ010916, GQ204308, GQ204309 and GQ204310).

Based on the conserved sequence of the known plant PCS proteins, four full-length cDNA clones encoding PCS in *S. rostrata*, designated *SrPCS1* (GenBank accession no. DQ010916), *SrPCS2* (GenBank accession no. GQ204308), *SrPCS3* (GenBank accession no. GQ204309) and *SrPCS4* (GenBank accession no. GQ204310), were isolated by RT-PCR amplification of conserved cDNA sequences combined with 3′ and 5′-rapid amplification of cDNA ends (RACE). The calculated molecular masses from the deduced amino acid sequences of *SrPCS1, SrPCS2, SrPCS3* and *SrPCS4* are 26 kD, 19.8 kD, 55.8 kD, and 19.9 kD, respectively (Figure 1).

Among the four *SrPCS* cDNAs, the ORF of *SrPCS3* is the longest one; its amino acid sequence shares 65% identity to *Arabidopsis* AtPCS1 [16], 87% identity to soybean GmhPCS1 [7] and 87% identity to *L. japonicus* LjPCS1 [18]. The identity of SrPCS3 with SrPCS1, SrPCS2 and SrPCS4 is 97.9%, 87.6% and 88.1%, respectively. The N-terminal domains of SrPCS3 and SrPC1, but not SrPCS2 and SrPCS4, contain a catalytic triad of Cys-56, His-162 and Asp-180 (Figure 1), which is essential for the activity [9,10,13,14].

**Figure 2.**
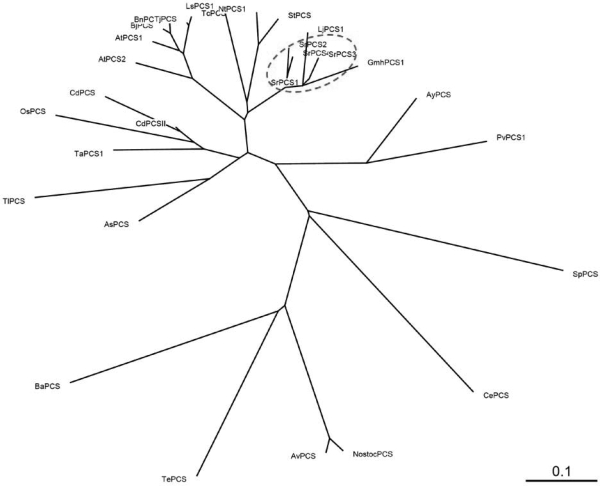
Phylogenetic analysis of PCS proteins from cyanobacteria, nematodes, fungi, and plants. The unrooted tree was constructed using the neighbor-joining method (ClustalW) with 1,000 bootstrp replicates. Branch lengths are proportional to genetic distance, which is indicated by a bar (0.1 substitutions per site). Abbreviations and GenBank accession numbers are as follows (in parentheses) AsPCS (*Allium sativum* AAO13809), AtPCS1 (*A. thaliana*, AAD41794), AtPCS2 (*A. thaliana*, AAK94671), AyPCS (*Athyrium yokoscense*, BAB64932), BjPCS (*Brassica juncea*, CAC37692), NtPCS1 (*Nicotiana tabacum,* AAO74500), TaPCS (*Triticum aestivum*, AAD50592), OsPCS (*Oryza sativa,* AAO13349), TjPCS (*Thlaspi japonicum*, BAB93119), TlPCS (*Typha latifolia*, AAG22095), SpPCS (*Schizosaccharomyces pombe*, CAA92263), StPCS (*Solanum tuberosum*, CAD68109), CdPCS (*Cynodon dactylon*, AAO13810), GmPCS (*Glycine max,* AAL78384), LjPCS1 (*Lotus japonicus*, AY633847), NsPCS (*Nostoc sp.* PCC 7120, NP-485018), SrPCS1 (*S. rostrata*, DQ010916), SrPCS2 (*S. rostrata*, GQ204308), SrPCS3 (*S. rostrata*, GQ204309), SrPCS4 (*S. rostrata*, GQ204310), PvPCS1 (*Pteris vittata*, AAT11885), CdPCSII (*Cynodon dactylon*, AAS48642), AvPCS (*Anabaena variabilis* ATCC 29413, YP_323464), TePCS (*Trichodesmium erythraeum* IMS101, YP_722155), BmPCS (*Burkholderia mallei* SAVP1, YP_993352), BnPCS (*Brassica napus*, CAK24968), TcPCS (*Thlaspi caerulescens*, BAB93120), and LsPCS1 (*Lactuca sativa*, AAU93349).

An unrooted phylogenetic tree (Figure 2) was constructed including the majority of the predicted PCS sequences available in the databases. The tree revealed separate clusters, paralleling taxonomic distance, for the PCSs of cyanobacteria, nematodes, yeast, ferns, and the families of vascular plants Poaceae, Allaceae-Typhaceae, Solanaceae, Brassicaceae, and Leguminosae. As shown in Figure 2, SrPCS proteins (in red dashed ellipse of Figure 2) are highly homologous to leguminous soybean GmPCS [7] and *Lotus* LjPCS1 [18]. The structure and function of *PCS* genes have only been studied in detail in the model plant *Arabidopsis*. Two functional PCS genes, *AtPCS1* and *AtPCS2*, which are localized in different chromosomes (At5g44070 and Atlg03980), were found in *Arabidopsis* [6,10,17,24]. In order to study the structure of *S. rostrata* PCS gene (*SrPCS*), we cloned the genomic sequence of *SrPCS* from the genomic DNA of *S. rostrata* by genome walking [28] with primers based on the conserved sequence of *SrPCS1-4* cDNAs. Unexpectedly, only a 5,112-bp genomic DNA fragment of *SrPCS* (GenBank accession no. GQ204307) was obtained.

### 2.2. Genomic Organization and Pre-mRNA Splicing of SrPCS Gene

**Figure 3.**
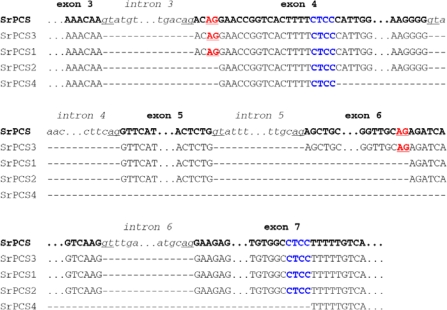
Alternative splicing of the pre-mRNA for the *SrPCS* gene. Exon nucleotides are shown as bold upper-case letters, and intron nucleotides are shown as italic lower-case letters. Canonical GT-AG junctions are underlined. Alternative AG acceptor splice sites are shown as bold upper-case red letters. Atypical splicing sites are shown as bold upper-case blue letters.

Alignment of the genomic sequence of *SrPCS* with those of four cDNA sequences of *SrPCS1-4* revealed that all four mRNAs (*SrPCS1-SrPCS4*) were produced from the one *SrPCS* gene through alternative splicing (Figure 3).

By comparing with the sequence of the canonically spliced form *SrPCS3* mRNA, eight exons and seven introns were found in *SrPCS* gene. As shown in Figure 3, *SrPCS1* and *SrPCS2* are generated through alternative AG acceptor splice sites. And the *SrPCS4* is produced by atypical splice mechanism employing direct repeats at splice sites (CTCC, Figure 3), which is similar to the atypical mRNA processing of p53 transcripts in the human brain [29].

**Figure 4.**
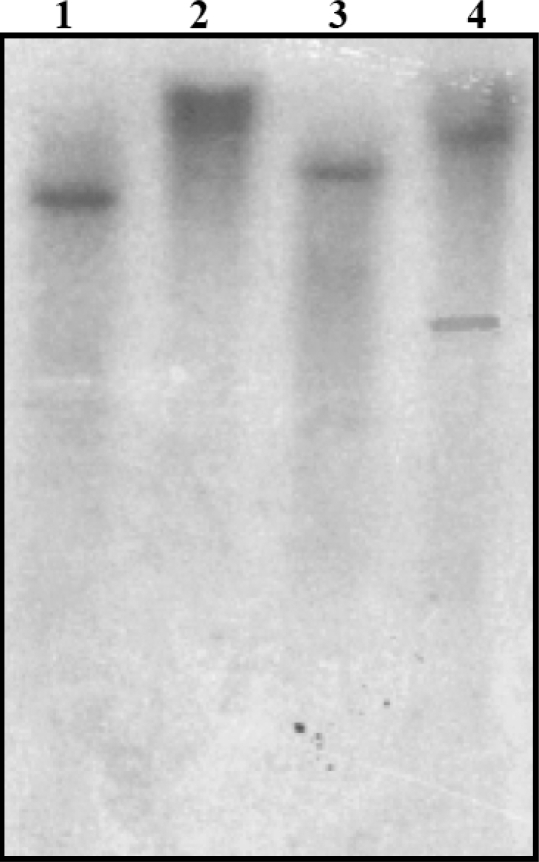
Southern blot analysis of *S. rostrata* genomic DNA. *S. rostrata* genomic DNA (25μg) digested with *Eco*RI (lane 1), *Bam*HI (lane 2), *Xba*I (lane 3), and *Pst*I (lane 4) was separated by agarose gel electrophoresis, blotted onto Hybond-N membrane and hybridized to the ^32^P-labeled conserved *SrPCS* cDNA fragment (nt 47-919 of GenBank GQ204309).

The presence of a single copy of the *SrPCS* gene in *S. rostrata* genome was further confirmed by Southern blot analysis (Figure 4). Only a single hybridizing band was detected in DNA samples digested with either *Eco*RI, *Bam*HI or *Xba*I, which do not cut within the probe region (Figure 4, lane 1–3). When the genomic DNA was digested with *Pst*I that cut once within the probe region, two hybridizing bands were observed (Figure 4, lane 4). These results indicate that the *S. rostrata* genome contains only a single copy of the *SrPCS* gene.

### 2.3. Expression Analysis of SrPCS Gene

Alternative splicing of pre-mRNA is often regulated by specific factors in response to developmental or environmental cues [30–32]. To find out whether the alternative splicing of *SrPCS* pre-mRNA is regulated by heavy metals, we investigated *SrPCS* expression in plants in the presence and absence of Cd^2+^. As in wheat [17] and *Brassica juncea* [33] plants, *SrPCS1*–*SrPCS4* mRNA expression was very low and could not be detected by RNA blot analysis of total RNA from different organs of *S. rostrata*. Therefore a more sensitive real time-PCR method was used to estimate the abundance of *SrPCS* transcripts. Unfortunately, because of very high sequence similarity of the four *SrPCS* mRNA, only specific primers for *SrPCS3* can be designed for real time-PCR analysis.

**Figure 5.**
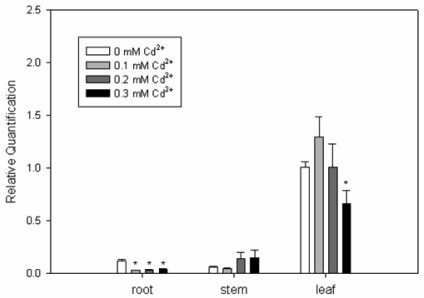
Expression analysis of *SrPCS3* transcript by real-time PCR. The relative expression of *SrPCS3* to an internal control glyceraldehyde-3-phosphate dehydrogenase (*GAPDH*) in *S. rostrata* plants, treated with different concentration of Cd^2+^ for 24 hours, was analyzed by real-time RT-PCR. Mean values plus standard errors are given. * indicates significance at the 5% level.

Unlike in Arabidopsis and *Brassica juncea*, where higher amounts of Arabidopsis *AtPCS1* transcript [6] and *B. juncea* BjPCS1 protein [33] were detected in roots than in leaves, *SrPCS3* is more highly expressed in the leaves than roots and stems under normal growth conditions without Cd^2+^ treatment (Figure 5). *SrPCS3* expression was significantly reduced in roots after exposure to different concentration of Cd^2+^ for 24 hours. No significant change of *SrPCS3* expression was observed in stems, whereas *SrPCS3* expression was also significantly down-regulated in leaves by 0.3 mM Cd^2+^ treatment. These observations are contrary to *TaPCS1* from wheat [17] and *LjPCSs* from the model legume *Lotus japonicus* [18]. Clemens *et al*. [17] found that *TaPCS1* showed a 5- to 10-fold higher expression in wheat roots treated with 0.1 mM Cd^2+^ for 6 h. Ramos *et al*. [18] investigated the expression of three PCS genes in roots of *L. japonicus*, and found that the mRNA expression of *LjPCS1-8R* increased only slightly (2.1-fold) after 6 h of 0.1 mM Cd^2+^ treatment, whereas the mRNA levels of *LjPC2-7R* and *LjPCS3-7N* increased 2.3- to 2.9-fold and 2.5- to 3.5-fold between 6 and 96 h, respectively.

### 2.4. Functionality of Four SrPCS Isoforms SrPCS1–SrPCS4

**Figure 6.**
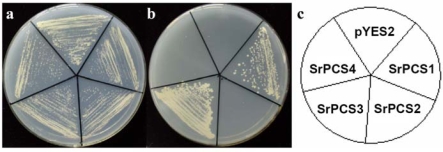
Cd tolerance assay of yeast cells expressing *SrPCS* cDNAs. Growth of control *S. cerevisiae* cells (carrying the empty pYES2 plasmid) and cells expressing one of the *SrPCS1–SrPCS4* on YNB plate without (a) and with 600 μM Cd^2+^ (b). (c) a diagram showing the cells on plates shown in (a) and (b).

**Figure 7.**
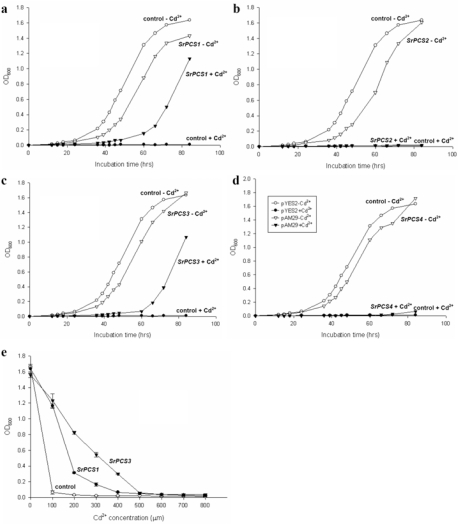
Growth curves of yeast cells expressing *SrPCS* cDNAs. Growth of control *S. cerevisiae* cells carrying the empty pYES2 plasmid and cells expressing *SrPCS1* (a), *SrPCS2* (b), *SrPCS3* (c), or *SrPCS4* (d) in YNB medium (supplemented with 1% raffinose + 1% galactose) containing either no (open symbols) or 200 μM Cd^2+^ (filled symbols). (e) growth of control *S. cerevisiae* cells carrying the empty pYES2 plasmid and cells expressing *SrPCS1* or *SrPCS3* in YNB medium (supplemented with 1% raffinose + 1% galactose) containing different concentrations of Cd^2+^. OD_600_ of cultures after 65 h is shown.

To test the functionality of four *SrPCS* isoforms, the full-length cDNAs of *SrPCS1–SrPCS4* were expressed in *S. cerevisiae* under the control of the inducible pGAL1 promoter. Cd tolerance assay showed that *S. cerevisiae* cells expressing *SrPCS1* or *SrPCS3*, but not *SrPCS2* and *SrPCS4*, exhibited strong tolerance to Cd^2+^ (600 μM), which completely inhibited the growth of cells transformed with the empty pYES2 plasmid or cells expressing *SrPCS2* or *SrPCS4* (Figure 6). Growth curve experiments (Figure 7, a–d) also demonstrated that expression of *SrPCS1* and *SrPCS3* conferred enhanced Cd tolerance to yeast cells (Figure 7, a and c), whereas the expression of *SrPCS2* and *SrPCS4* did not make yeast cells more Cd tolerant (Figure 7, b and d).

Dose-response analyses showed that *SrPCS3*-expressing yeast cells tolerated higher Cd^2+^ concentrations than the *SrPCS1* expressing cells and control cells (Figure 7, e), which may result from the lack of the C-domain that has been shown to ensure higher PCS activity [11]. Alternative splicing, unlike gene promoter activity that primarily regulates the amount of transcripts, changes the structure of transcripts and their encoding proteins [34]. Therefore alternative splicing is one of the most important mechanisms to generate a large number of mRNAs and protein isoforms from a single or few genes [35]. Several studies have shown that about 20% of plant genes have one or more alternative transcript [36–39]. And based on the alignments of ESTs from other legume species against the *Medicago truncatula* genome sequence, hundreds of legume genes were recently predicted to be alternatively spliced [39].

In this study, we found that a single copy of the *SrPCS* gene occurs in *S. rostrata* genome, and that alternative splicing of the *SrPCS* pre-mRNA produced four different *SrPCS* mRNAs and proteins, SrPCS1–SrPCS4 (Figure 1). The SrPCS1 and SrPCS3 proteins, whose N-terminal domains contain a catalytic triad of Cys-56, His-162 and Asp-180 (Figure 1), conferred Cd tolerance when expressed in yeast cells (Figure 6 and 7), whereas the SrPCS2 and SrPCS4 proteins, which contain only a Cys-56 of the catalytic triad and lack the N-terminal domains (Figure 1), did not render yeast cells more Cd tolerant (Figure 6 and 7). This observation is similar to the findings of Ramos *et al*. [18], who showed that a PCS gene (*LjPCS2*) in model legume *Lotus japonicus* can be alternatively spliced to encode two protein isoforms, one (LjPCS2-7N) conferring enhanced Cd tolerance to yeast cells while the other (LjPCS2-7R) did not. SrPCS1 and SrPCS3 characterized in this study have potential applications in genetic engineering of plants for enhancing heavy metal tolerance and phytoremediation of contaminated soils [40]. Although we detected the mRNA expression of the four SrPCS isoforms in the current study, the corresponding proteins of these mRNAs and their physiological functions remain to be characterized. In particular, the presence of SrPCS2 and SrPCS4 proteins, which lack the catalytic triad and the N-terminal domains and do not confer enhanced Cd tolerance to yeast cells, needs to be determined in *S. rostrata* plants, since they are small variants of the full-length protein (about one-third of the SrPCS3) and may not be stable if translated in plant cells.

## 3. Experimental Section

### 3.1. Plant Materials

Seed testae of *Sesbania rostrata* were scalpelled and surface-sterilized in 70% alcohol for 1 minute, followed by three rinses with sterile distilled water, and then soaked in 10% sodium hypochlorite for 15 minutes with gentle agitation. After washing three times with sterile distilled water, the seeds were germinated on 1/2 MS medium [41] for 6 days. These seedlings were then transferred to 1/8 liquid MS without sugar to grow in this medium for 15 days, changing medium every three days, and then transferred to 1/4 liquid MS containing different concentration of cadmium for 1–2 days as needed at 24 ± 2 °C under fluorescent light with a 16-h photoperiod.

### 3.2. Cloning of PCS cDNAs and Gene from S. rostrata

Total RNA was extracted from seedlings treated with Cd for 2 days as described above using the Trizol reagent (Invirtogen). The cDNA was synthesized from 2 μg of total RNA that was treated by DNaseI before reverse transcription (RT) with the M-MLV (Progema). RT-PCR amplification of the conserved partial sequence of *SrPCS* cDNAs was performed with a pair of primers (forward primer ZF168, 5′-GCGTTAAGAGACAATGGCGATG-3′; reverse primer ZF169, 5′-GGGAGATGCTAAG-AAAAGGGTACA-3′) based on the sequence of soybean PCS [7]. The amplification parameters were as follows: 5 min at 95 °C, 30 cycles of 40 s at 95 °C, 40 s at 54 °C and 2 min at 72 °C, 10 min at 72 °C in a 20-μl reaction mixtures. The 5′- and 3′- ends of *SrPCS* cDNAs were isolated with a modified RACE procedure based on template-switching effect and inverse PCR [42]. Genomic DNA from *S. rostrata* was extracted by a modified CTAB method [43]. *SrPCS* genomic clone was obtained using the Universal GenomeWalker Kit (Clontech) according to the manufacturer's protocol. PCR products were cloned into pGEM-T easy (Progema) and sequenced. Sequence identity was calculated by the Ident and Sim program in the Sequence Manipulation Suite (http://www.bioinformatics.org/sms/).

### 3.3. Real Time RT-PCR of SrPCS3

Real time RT-PCR was used for the quantification of the *SrPCS3* expression using the Premix Ex Taq^™^ (Perfect Real Time, TakaRa) with a pair of *SrPCS3* specific primers (forward primer ZF332, 5′-GCTTTATACTCTGAGCTG-3′; reverse primer ZF335, 5′-TTAGATGGCAG-TGATGT-3′). As an internal control, glyceraldehyde-3-phosphate dehydrogenase (*GAPDH*) mRNA was simultaneously amplified to normalize *SrPCS3* expression with a pair of *GAPDH* specific primers (forward primer ZF371, 5′-GCTGGTGCTGATTATGTT-3′; reverse primer, ZF372 5′-GCTCTGGCTTGTATTCCT-3′). PCR cycling conditions were: 15 s at 95 °C; 40 cycles of 5 s at 96 °C, 20 s at 58 °C and 30 s at 72 °C in a 25-μl reaction mix containing 1x SYBR Green I. Each sample was performed in triplicates at least three times. Amplification products were measured and analyzed with an ABI Prism 7900HT sequence detection system and SDS software (Applied Biosystems).

### 3.4. Southern Blot Analysis of SrPCS

For Southern blot analysis, 25 μg genomic DNA was digested with restriction enzymes and separated on 0.8% agarose gel and transferred to Hybond-N membrane (Amersham) according to Sambrook and Russell [44]. The conserved *SrPCS* cDNA fragment (nt 47-919 of GenBank GQ204309) was labeled with [α-^32^p] dCTP using Random Primer Kit (TakaRa) and used to detect DNA on the membrane as previously described [45].

### 3.5. Expression of Recombinant SrPCS1-4 in Yeast and Cd Tolerance Assay

Four ORFs of *SrPCS1-4* cDNAs were amplified by PCR with a pair of *SrPCS*-specific primers carrying a *Bam*HI and a *Kpn*I restriction enzyme site (forward primer ZF268, 5′-GGTACCATGGCG-ATGGCGGGGTTGTA-3′; reverse primer ZF269, 5′-ATGGGATCCGGAGATGCTAAGAAAAGG-3′), respectively. After digestion with *Bam*HI and *Kpn*I, each of four PCR fragments containing the ORF of *SrPCS1-4* was ligated in the yeast expression vector pYES2 (Invitrogen), resulting in four recombinant vectors (pAM26, pAM27, pAM28, and pAM29) that were used to transform *Saccharomyces cerevisiae* strain INVSc1 (MATK his3v1 leu2 trp1-289 ura3-62) cells according to the supplier’s protocol (Invitrogen). For the Cd tolerance assay, yeast cells containing the empty vector pYES2 or the *SrPCS1-4* cDNA constructs were grown in the yeast nitrogen base (YNB) medium supplemented with the appropriate amino acids, 1% galactose and 1% raffinose containing different concentrations of Cd^2+^ at 30 °C.

## 4. Conclusions

Our results demonstrate that *S. rostrata*, a tropical legume plant tolerant to heavy metals, contains a single copy of the *SrPCS* gene in its genome, which produces four mRNAs encoding different protein isoforms (SrPCS1–SrPCS4) by alternative splicing of the *SrPCS* pre-mRNA. When expressed in yeast cells, the SrPCS3 protein containing the catalytic triad and the N-terminal domain rendered yeast tolerant to higher Cd^2+^ concentrations than the SrPCS1 protein containing the catalytic triad but lacking the N-terminal domain. These results suggested that *SrPCS1 and SrPCS3* could be used for the production of heavy metal tolerant transgenic plants. The other two alternatively spliced variants, SrPCS2 and SrPCS4, which lack the catalytic triad and the N-terminal domains and do not confer enhanced Cd tolerance to yeast cells, may play different roles in *S. rostrata* growth and development, although the presence of these two proteins in plants needs to be confirmed.
